# Identification of Diptera Puparia in Forensic and Archeo-Funerary Contexts

**DOI:** 10.3390/insects15080599

**Published:** 2024-08-08

**Authors:** Stefano Vanin, Fabiola Tuccia, Jennifer Pradelli, Giuseppina Carta, Giorgia Giordani

**Affiliations:** 1 Department of Earth, Environmental and Life Sciences (DISTAV), University of Genoa, 16132 Genoa, Italy; giuseppina.carta@edu.unige.it; 2 National Research Council, Institute for the Study of Anthropic Impact and Sustainability in the Marine Environment (CNR-IAS), 16149 Genova, Italy; 3 School of applied Sciences, University of Huddersfield, Huddersfield HD1 3DH, UK; tuccia.fabiola@gmail.com (F.T.); jennifer.pradelli@gmail.com (J.P.); 4 Independent Researcher, 40126 Bologna, Italy; giorgia.giordani.gg@gmail.com

**Keywords:** forensic entomology, bioarcheology, zooarcheology, species identification, Diptera, puparia, molecular identification, cuticular hydrocarbons, cuticular lipids

## Abstract

**Simple Summary:**

In forensic entomology, insects are used to estimate the time since death and to answer other investigative questions, such as determining if the body was moved postmortem or identifying the season of death. In funerary archeoentomology, insects provide information related to the season of death, body transfer, and funerary practices. To obtain this information, the correct identification of specimens collected from the scene is fundamental. In old cases and archeological contexts, the majority of entomological evidence samples found are fly puparia, barrel-like structures formed from the third-instar larval cuticle where metamorphosis takes place. Despite recent publications of identification keys for certain fly larvae and adults, there is still a lack of tools for puparium identification. This problem is exacerbated by the scarcity of diagnostic features present on the puparia. This paper critically reviews the techniques available for puparium identification in addition to traditional morphological analysis. It describes DNA-based approaches and chemical analyses of cuticular lipids and hydrocarbons, highlighting the importance of using non-invasive methods to guarantee the repeatability of the analysis—a key element in forensic science—or to preserve precious and unique specimens from museum collections.

**Abstract:**

Diptera identification is fundamental in forensic entomology as well as in funerary archeoentomology, where the challenge is exacerbated by the presence of immature stages such as larvae and puparia. In these two developmental stages, specimens possess a very limited number of diagnostic features, and for puparia, there is also a lack of identification tools such as descriptions and identification keys. Morphological analysis, DNA-based techniques, and cuticular chemical analyses all show good potential for species identification; however, they also have some limitations. DNA-based identification is primarily hindered by the incompleteness of genetic databases and the presence of PCR inhibitors often co-extracted from the puparial cuticle. Chemical analysis of the cuticle is showing promising results, but this approach is also limited by the insufficient profile database and requires specific, expensive equipment, as well as trained personnel. Additionally, to ensure the repeatability of the analysis—a critical aspect in forensic investigations—and to preserve precious and unique specimens from museum collections, non-invasive protocols and techniques must be prioritized for species identification.

## 1. Introduction

In a forensic context, insects as other environmental living organisms (e.g., microbes, algae, plants, etc.) or their fragments (e.g., leaves, open puparia, etc.) can provide useful information to answer the investigative questions—summarized in English as the 6Ws (When, Where, What, How, Who and Why) [1]. In particular, forensic entomology is the application of the knowledge about insects and other arthropods in legal matters and it has been developed, since the end of the 19th century, mainly to estimate the time since death, or better the minimum Post Mortem Interval (min PMI) [2,3]. Moreover, in the last century, due to the increase in the knowledge about insect phenology, habitat and distribution, the development of new technologies and analytical methods insects have been used also to answer other questions such as the season of death, the primary location of the body (the primary crime scene), the presence of drugs and also the DNA identification of the victim in absence of the body if larvae or puparia are still present in the crime scene [4,5,6]. This approach has also been successfully applied with beetle frass [7].

Funerary archeoentomology shares the same bulk of knowledge related to the insect colonization of human and animal bodies, and some common techniques for the collection and analysis of the samples with forensic entomology; however, the two disciplines are separate and well distinct.

The main pillars on which forensic entomology is structured may be summarized in four points:

1. Insect development is temperature-dependent, species-specific, and population-related. This allows the estimation of the minPMI for short periods of time if the temperature experienced by the first colonizers is known [8,9].

2. The order in which insects colonize a body is well defined and, in some ways, predictable [2]. This allows the estimation of the minPMI for long periods based on the entomo-community present on the body/remains.

3. Habitat and phenology are characteristics of each species. These allow the estimation of the season of death in old cases and the evaluation of a body transfer after the death. This point has been discussed by several authors (e.g., [10]) and it has been indicated that the structure of the landscape also has to be considered because it plays a fundamental role in species distribution [11]. It is also worth mentioning that climate change is strongly affecting species phenology and distribution [12,13], with a longer period of activity of the species in the cooler seasons or with bimodal phenologies. In addition, recent species invasions to distant regions makes global carrion entomofauna, especially key forensically important species, particularly blowflies, house flies, and flesh flies, highly similar/homogenous.

4. The effect of drugs on insect development depends on the species, with some species more affected, positively or negatively, in terms of the rate of development, and other species less or not affected [4]. This observation makes clear the need for a complete knowledge of the effects of different kind of drugs on different species.

From these points, it is evident that the beginning of any entomological investigation, not only in the forensic field but also in other fields such as those that are medical, agricultural, archeological, etc., must begin with the correct identification of the species and their developmental stage. This “*conditio sine qua non*” can theoretically be satisfied, as any entomologist and zoologist knows, through the comparison of specimens with the *typus* stored in a public collection as reported by the International Code of Zoological Nomenclature [14]. This process typically works well when an adult specimen is considered because species are usually described based on adult specimens. However, a practical approach is the use of dichotomous identification keys for identifying specimens. Again, this approach works quite well when adults are considered.

**Figure 1:**
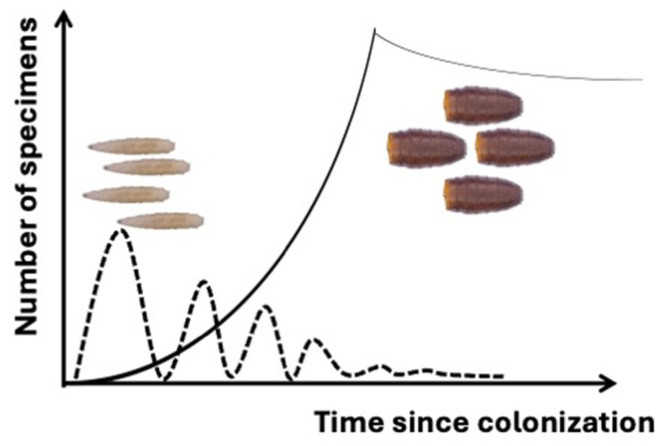
Schematic representation of the trend in the number of larvae (dashed line) and puparia (continuous line) since the time of colonization. The slow decrease in the number of puparia with time is related to predation, dispersion, and taphonomic processes.

In forensic entomology, the majority of the samples collected by crime scene investigators—entomologists who are rarely involved in the crime scene survey—or during autopsies by coroners or forensic pathologists, depending on the legal system, are not composed of adults but of immature stages such as larvae and puparia, or, in old cases and in archeo-funerary contexts, traces of their previous presence (e.g., empty puparia, exuviae, cocoon, frass). It is worth mentioning that the number of fly larvae will fluctuate during the colonization process, whereas the number of puparia will progressively increase until the last larva has pupated (Figure 1). The slow decrease in the number of puparia with time is related to predation, dispersion, and taphonomic processes.

In recent years, identification keys for certain larvae have been developed [15,16,17,18,19] and in Europe, most larvae of Calliphoridae, as well as some genera of Fanniidae and Muscidae that are of forensic interest, are relatively easy to identify. However, the situation is quite different when considering puparia. Despite a few older works [20], scattered descriptions in the general literature [2], and only a handful of recent publications [21,22,23,24,25], there remains a significant gap in knowledge regarding the puparia of the majority of species of forensic interest. Additionally, there is a scarcity of research describing the puparia of other families (e.g., [26]). When morphological characters are not readily detectable or are absent, the most common alternative approach for identification is molecular, based on DNA analysis [27,28]. Furthermore, in recent years, cuticle chemical analysis has shown promising results, although it does not currently cover all species of forensic interest [29].

Despite various suggested approaches for puparium identification, there remains an evident lack of structured and well-defined methods. For this reason, this paper aims to critically summarize the most commonly used techniques for puparium identification.

## 2. Definition, Structure, and Formation of Puparia

A detailed and precise description of the pupariation process with a revision in the nomenclature was provided by Martin-Vega and colleagues in 2016 [30]. In cyclorrhaphous flies, the pupal stage and the subsequent development of the pharate adult occur inside an opaque, barrel-like puparium formed from the cuticle of the third instar larva. This period, from puparium formation until adult emergence, comprises more than 50% of the total immature development, and once formed, the puparium does not exhibit external age-related changes [29]. Furthermore, puparia recovered from a body or a crime scene represent the only direct trace of a species’ presence after adult emergence and dispersal. Due to the chemical stability of chitin, puparia may survive for centuries or millennia [31]. Depending on the different phases of development occurring inside the puparium, the puparium can be closed (prepupa–pupa–pharate adult) or open after adult emergence. Several works in the forensic entomological field erroneously use the term “pupa” to describe the closed puparium. However, the term “pupa” should be avoided as it specifically refers to a developmental phase occurring inside the puparium and only for a limited time [20], and does not consider the prepupa and the pharate adult.

## 3. Morphology-Based Approach

**Figure 2:**
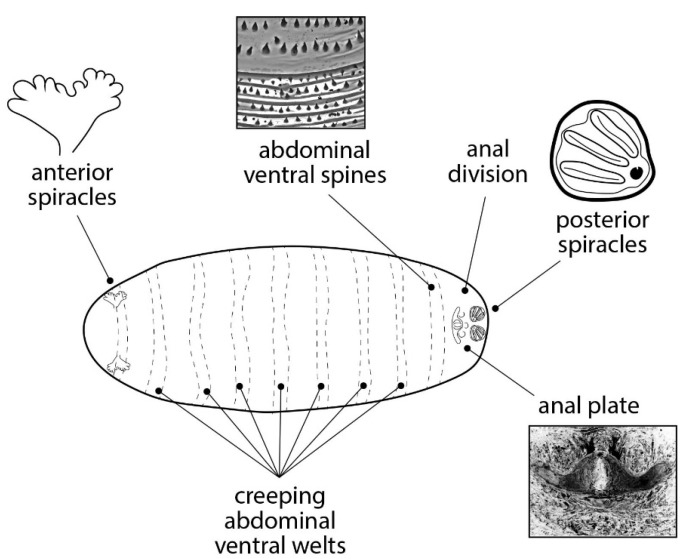
Diagnostic characteristics of Diptera puparia. Ventral views and anatomical details of a puparium analyzed for the diagnosis of the species are schematically shown. Each element is reliably reproduced from photographs; however, the whole representation is not realistic as the elements are not in scale and have been selected from multiple species.

The study of morphology is a well-established, traditional, and straightforward method of identification. This method involves observing diagnostic anatomical traits of interest and comparing them with published morphological descriptions or using identification keys. Regarding Diptera which are of forensic interest, available data primarily focus on certain groups of flies (e.g., Calliphoridae, Muscidae, and Piophilidae) for which ample information is available for adult specimens. However, immature stages often lack exhaustive descriptions. Difficulties arise primarily due to the few diagnostic characteristics exhibited by eggs, larvae, and puparia (Figure 2), and the fact that these features may not be equally useful for distinguishing different species [21,24].

Moreover, while it is common for puparia to retain the same characteristics as those of the larval stage [17,32], physical modifications such as contraction during pupariation may occur, resulting in a partial loss of detailed definition [30]. Additionally, elements such as oral sclerites are not always present among the remains of empty puparia, especially in old cases and in archeological contexts where puparia often represent the majority of Diptera findings. In these cases, fragments of adults are only rarely found [33] and when are found, they are in a bad state of preservation, with an important loss of diagnostic characteristics such as the setae, parts of the legs segments, antennae and palpi.

Particularly, in such cases and in certain families, the lack of retention of the anterior part of the puparium after adult emergence, where oral sclerites are conserved, makes species-level identification difficult, if not impossible. It is also noteworthy that while some characteristics may provide diagnostic information for certain taxa, they may not be informative for others. For example, in the forensic context, posterior spiracles are significant in blow flies (Calliphoridae) [17] and in house flies of the genus *Hydrotaea* Robineau-Desvoidy, 1830 (Muscidae) [21], but they offer poor information in Piophilidae, where anterior spiracles are more informative, at least at the genus level [24].

Breeding specimens has been proposed as a solution to overcome the challenge of puparium identification. However, this method is effective only when the organisms inside the puparia are alive, collected, and transported to the entomological lab in an appropriate way not affecting their survival. Unfortunately, this approach is not feasible when dealing with empty puparia or when laboratory rearing of the immature ones fails [34]. In such circumstances, it becomes crucial to compare insect remains to specimens in private or natural history museum collections and to collaborate with taxonomy experts to successfully identify a particular species [35,36,37]. Alternatively, a DNA-based approach can be pursued.

In addition, global warming and globalization are affecting the species distribution with several allochthonous species of forensic interest already introduced in new areas [13,38]. For example, in Europe, *Chrysomya megacephala* (Fabricius, 1794) has been reported in Portugal, Spain, and Malta [39,40,41], and *Synthesyomya nudiseta* Van Der Wulp, 1883 has also been reported in Italy from human cadavers [12]. In order to be recognized and identified, a newly introduced species requires detailed knowledge of more than just the local entomofauna. In addition, it also requires the availability of general identification keys at a family or genus level rather than being focused solely on the species present at a regional scale. This requires the creation/formation/education of high-level taxonomists with a general knowledge of the taxa, a comprehensive understanding of their group of interest’s taxa, and not just knowledge limited to the local fauna or specific geographical subjects. Unfortunately, it appears that research grants are not focused on this direction. 

In the context of Europe, the problem of the shortage of taxonomists is well-documented in the European Red List of Insect Taxonomists [42]. Based on a quantitative analysis of taxonomic papers published in scientific journals over the last decade, as well as an online questionnaire, the red list provides an overview of the taxonomic capacity for each insect order and EU country. The red list highlights that the taxonomic capacity is threatened or eroded for 41.4% of the insect orders at the European level. Additionally, the four largest insect orders (Coleoptera, Diptera, Lepidoptera, and Hymenoptera), which include insects of forensic interest, are covered by over 80% of the countries (even over 90% for Coleoptera and Lepidoptera). However, the adequate capacity to identify insects, describe, and name new species is only achieved in 26% (Coleoptera) to 58% (Hymenoptera) of all countries. Unfortunately, Diptera are listed as a taxon with a poor capacity at the European level. Despite the lack of official studies, to the authors’ knowledge, it seems that the European taxonomist situation is the same, if not worse, on the other continents.

Universities, museums, and institutes dealing with forensic entomology have a significant responsibility in addressing this issue. They should be supported at the international, national, and local levels to enhance their taxonomic capacity, particularly in the main groups of forensic interest. This support should not be limited exclusively to the six main families of flies (Calliphoridae, Sarcophagidae, Muscidae, Fanniidae, Phoridae, and Piophilidae) or beetles, but should also involve dedicated knowledge exchange, education, training, and opportunities for professional taxonomists. Finally, local and global governments have to take into account the need for making available adequate resources for the perpetuation of taxonomic expertise that, in some cases, can lead to the discovery and prevention of human and veterinary health issues, such as in the cases of vector-borne diseases that account for more than 17% of all infectious diseases, causing more than 700,000 deaths annually.

## 4. DNA-Based Approach

Molecular identification has become a widely used tool to support traditional morphological studies in corroborating species identification [43,44]. Insects can potentially be unequivocally identified by sequencing a distinctive DNA region known as the “barcode” [45]. The mitochondrial COI gene has been selected as the molecular target for distinguishing several metazoan species, as it exhibits the highest pairwise inter-specific divergence and the lowest pairwise intra-specific divergence, defined as the frequency of mutations between two DNA sequences [46]. Mitochondrial targets are present in high copy numbers in cells and demonstrate a higher mutation rate compared to nuclear targets. The COI gene (1535 bp in its entirety) encodes for subunit I of Cytochrome Oxidase, a protein involved in the electron-transport chain of cellular respiration. However, only the 658 bp at the 5′ end of the gene have been designated as the “barcoding region”, which can be amplified using “universal primers” [47]. Furthermore, new sets of primers annealing within or between the barcoding region have been designed and recently reviewed [43]. However, while shorter barcode sequences, as suggested in general for metazoan taxa by Yeo and colleagues [48], have proven successful in identifying Fanniidae of forensic interest [49], DNA fragments shorter than ~200 bp are not recommended for identifying Sarcophagidae species [50]. The use of multiple primer pairs, referred to as “mini barcoding”, allows for the amplification of overlapping fragments that can be aligned through in silico analysis, generating a longer nucleotide sequence. This approach is advantageous for increasing amplification success when DNA is highly degraded and in cases of ancient samples.

In cases where closely related species exhibit low inter-specific divergence, COI analysis may provide unreliable discrimination, as seen in the case of *Lucilia caesar* (Linnaeus, 1758) and *Lucilia illustris* Meigen, 1826 (Diptera: Calliphoridae) [51]. In such instances, alternative genetic targets can be utilized, including mitochondrial targets such as COII (cytochrome c oxidase subunit II) [52], Cytb (cytochrome b) [46,53,54], ND5 (NADH dehydrogenase 5) [55,56], and 28S rDNA (28S ribosomal subunit) [57], or nuclear targets such as EF-1a (elongation factor 1a) [56,57], ITS1 (internal transcribed spacer 1) [46,51], ITS2 (internal transcribed spacer 2) [58], and PER (period) [56,59]. The combined use of these targets, known as “concatenation” [56], is another option to improve the resolution of closely related species. However, in recent years, whole genome sequencing has provided the best results in resolving sibling species such as in the genus *Lucilia* Robineau-Desvoidy, 1830 [60].

The primary advantage of using genotyping for identification purposes is its applicability to any specimens, regardless of their developmental stages. The approach is well-established for adults and larvae of all instars, but further investigation is needed to routinely apply it to puparia. This limitation is related to the amount of extractable DNA, as the epithelial tissue (larval exuviae) that separates the metamorphic fly from the puparium until the transformation is complete serves as the only available source of DNA. In 2010, Mazzanti and colleagues reported the successful identification of 42 Diptera puparia collected from crime scenes, with the oldest dating back to 20 years before DNA extraction [28].

While laboratory procedures for identifying insect species are well-established, there are several pitfalls related to data interpretation, which is a crucial step in every scientific investigation [27]. Worldwide researchers generate nucleotide sequences that populate reference DNA databases, such as GenBank at the National Center for Biotechnology Information (NCBI) and the European Bioinformatics Institute (EBI), which include information for over 100,000 Eukaryotic species. However, the submission of DNA sequences to the database is open to all users and is not subject to any request for proof of specimen identification. This lack of compliance with standard guidelines established by taxonomically oriented databases can result in inaccuracies [61]. Users are implicitly expected to ensure that only high-quality, contamination-free sequences populate the databases. In 2003, Tautz proposed the idea of a taxonomically oriented database where data could be subject to innovative management to ensure their authenticity and traceability, which was later realized with the Barcode of Life Data System (BOLD), a repository of mtCOI barcoding sequences only [62].

Regarding species of forensic interest, significant research has been dedicated to the most common taxa, especially in the families of Calliphoridae, Sarcophagidae, and Muscidae, whose DNA sequences largely populate DNA databases. However, little effort has been directed towards less common taxa, including Muscidae, Phoridae, and Piophilidae, for which only limited datasets of sequences are available. For these reasons, molecular phylogenetic analysis should be carefully considered and interpreted. Wells and Stevens [27] provided a detailed review of these aspects, which constitute the “other side of the coin” of molecular analysis applied to species identification in forensic entomology.

Furthermore, puparia often represent the majority or the only entomological evidence in old cases, and DNA analysis presents similar challenges to what can be considered ancient DNA due to the low quantity, potential degradation, and presence of PCR inhibitors.

### 4.1. Fragmentation and Chemical Modifications

DNA retrieved from old and ancient substrates can be highly fragmented [37,63,64,65,66,67,68,69,70,71,72]. Several factors contribute to the “natural” or induced breakdown of the double helix. The collapse of cellular structures exposes nuclear contents to attacks from biotic and abiotic environmental factors, including exogenous nucleases [71,73] released by microbial communities spreading and feeding on dead tissues [74]. Additionally, exposure to UV light induces double-strand breaks in DNA molecules [75]. Under specific circumstances, such as rapid desiccation and low temperatures, fragmentation might be reduced, but chemical modifications may occur and further destabilize the DNA backbone. The oxidation of nitrogenous bases, hydrolytic processes of deamination or depurination, and cross-link formation with other molecules are common damages contributing to DNA decay [68], leading either to the rupture of DNA molecules or rendering them inaccessible to DNA polymerase during PCR [64].

### 4.2. PCR Inhibition

PCR inhibition is another challenge. Mitochondrial genes such as COI, COII, 16S rDNA (16S ribosomal subunit) (present in thousands of copies per cell), and nuclear genes like 18S rDNA (18S ribosomal subunit) or ITS2, organized as tandem-repeat sequences within the nuclear genome, are preferred targets [64]. However, even with a high probability of success in the planned strategy, other exogenous mechanisms of inhibition may occur at any point during molecular interactions during amplification. One of the most common origins of PCR inhibitors is compounds co-extracted with DNA, often resulting in impure DNA samples if not sufficiently purified. The exact molecular mechanism is poorly understood, but it is speculated that these compounds can form high-molecular-weight complexes with the template or with DNA polymerases, thus sequestering the substrate from the enzyme or vice versa [73]. Moreover, some compounds can chelate Mg^2+^, which is the essential cofactor of DNA polymerases. Although predicting the nature of inhibitors affecting environmental samples is difficult, a list of compounds that can be co-extracted with host DNA includes polysaccharides, humic and humic-like compounds, other polyphenolic compounds, fulvic acids, and heavy metals [73]. Interestingly, Pedersen and colleagues [71] note that humic acids bind to DNA molecules due to their negative surface charge, allowing the formation of inhibitory molecular complexes. However, it is through the formation of such complexes that ancient DNA can effectively survive for prolonged periods. To address this issue when working with environmental samples, the addition of compounds such as PVP (polyvinylpyrrolidone) or PVPP (polyvinylpolypyrrolidone) and BSA (bovine serum albumin) to PCR has been proposed as a solution. These compounds have a higher affinity for inhibitors than for the reaction components effectively inhibited (DNA template or DNA polymerases) [66,76,77].

## 5. Chemical Identification of Puparia

Over the years, alternative approaches to traditional morphological examination and DNA-based analysis have emerged for the identification of blowfly puparia species. These methods involve the analysis of chemical profiles, which can be easily conducted using instruments commonly found in forensic laboratories. Not only are these chemical analyses more objective, but they are also subject to empirical scrutiny and can provide information about their own margin of error.

Among the various proposed methods of analysis, one idea has shown great promise. It involves analyzing the organic compounds extracted from the insect cuticle. The cuticle is associated with a wide range of chemicals including hydrocarbons, free fatty acids, alcohols, aldehydes, wax esters, and fatty acid methyl esters. These compounds play different roles in the insect’s physiology and life cycle [78]. 

### 5.1. Cuticular Hydrocarbons

Cuticular hydrocarbons (CHCs) are constituents of the lipid wax layer found on insect cuticles. They consist of long linear chains of hydrogen and carbon atoms with a chain length ranging from C17 to C35. In insects, CHCs can exist in saturated and unsaturated forms and may contain one or more methyl groups.

Previous research on CHCs in various insects has demonstrated their efficacy as identification tools and aging indicators [79]. Studies on forensically important blowflies have also shown promising results. Specifically, the analysis of CHCs using Gas Chromatography–Mass Spectrometry (GC–MS), non-metric multidimensional scaling analysis (NMDS), and permutational multivariate analysis of variance (PERMANOVA) for data interpretation appears to be an accurate method for identifying specimens from forensic cases in Europe [29].

However, several factors hinder the routine application of this technique:

- The lack of a comprehensive database of fly puparia CHC signatures;
- Changes in puparia CHC signatures over time, which, while useful for aging puparia, may affect identification precision;
- The requirement for specific and expensive equipment, as well as trained personnel, further complicates widespread adoption.

### 5.2. Lipidis

As described in several studies [78,80,81], flies contain a diverse array of fatty acids and sterols. A spectrum of free fatty acids has been identified, ranging from hexanoic acid (6:0) to hexacosanoic acid (26:0), although many of these are at trace levels compared to palmitic acid, palmitoleic acid, oleic acid, linoleic acid, and stearic acid. These lipids, like hydrocarbons, provide resistance to desiccation, fungal infection, and bacterial infection.

The chemical profiles may be useful as a tool for identification and discrimination at the puparium level, as these compounds exhibit qualitative and quantitative differences among species. Nevertheless, larvae and pupae have different chemical profiles, and so do pupae of different ages [82]. Although some papers have shown the discriminating power of chemical profiles for blowfly larvae and pupae under lab-standard conditions [79,80,83], these same studies also demonstrated that abiotic factors such as temperature, humidity, pupation substrate, and diet greatly reduce the accuracy of species identification using their chemical profiles.

## 6. Methodology Remarks

As discussed by some authors [44,84], for both larvae and adult insects, the possibility of repeating molecular and morphological identifications of Diptera specimens, from contemporary and old cases, is important in the forensic field. This is also crucial for the preservation of specimens stored in museum collections. For this reason, non-invasive molecular and morphological analyses should be prioritized.

Proper preparation of the specimens, such as dry mounting or storing them in ethanol (70–95%, room temperature) [34], and the use of immersion in DNA extraction buffers without smashing the specimens, are among the procedures that can be used to preserve the specimens and allow subsequent analyses, at least at the morphological level. Storage at −20 °C can be an optimal solution; however, it can be performed only for a short time or for dedicated specimens, depending on the available freezing facilities and their constant electricity supply. Storage at −80 °C does not improve the preservation of specimens and can affect DNA stability. The issue of repeatable molecular analysis may not be a concern when large numbers of specimens are found, especially if they exhibit unique diagnostic features. 

## 7. Conclusions

Due to the key importance and complexity of identifying immature Diptera in forensic entomology and funerary archoentomology, it is recommended to combine different methods after evaluating their potential and limitations. This evaluation should also consider the available equipment and the training and background of the personnel. Additionally, non-invasive analysis protocols and techniques should be prioritized.

A collection of previously identified specimens is a valuable tool, as it allows for direct comparisons of samples. Limitations in DNA sequence availability can also be overcome by creating lab databases with local species and uploading them to public DNA banks.

Chemical cuticular analyses are also quite valuable. However, the requirement for specific and expensive equipment, as well as trained personnel, complicates the widespread adoption of this approach in existing forensic entomology laboratories.

## Data Availability

All the data have been acquired from the available scientific literature as reported in the References. No other data have been generated.
